# In Vitro Veritas

**DOI:** 10.3389/ftox.2020.00001

**Published:** 2020-03-12

**Authors:** Mathieu Vinken

**Affiliations:** Department of In Vitro Toxicology, Vrije Universiteit Brussel, Brussels, Belgium

**Keywords:** *in vitro*, toxicology, cell culture, 21st century toxicity testing, mechanisms

In 1959, British scientists William Russell and Rex Burch published their seminal book “*The principles of humane experimental technique*,” which introduced the so-called 3Rs concept. In essence, this concept calls for refinement, reduction, and replacement of animal experimentation (Russell and Burch, 1959). Ethical awareness regarding the use of animals for scientific goals and other purposes, including regulatory safety evaluation of chemicals, has strongly increased since then, not only in animal welfare groupings, but equally throughout society as a whole. In fact, these ethical constraints have instigated the introduction of specific legislations in Europe, including the directive on the protection of laboratory animals for scientific purposes^1^. In the case of cosmetic products and their ingredients, the European legislator has even completely banned the use of animals for safety evaluation practice through the cosmetics regulation^2^, and this is becoming increasingly followed by other countries worldwide^3^. In addition to the ethical concerns, animal models have also been heavily criticized over the past decades because of their poor predictive capacity for the human situation. For instance, preclinical animal models only allow to predict 50–60% of human drug-induced liver injury cases because of interspecies differences (Laverty et al., 2010).

Altogether, these developments have triggered a major paradigm shift in the field of toxicology and chemical safety evaluation, which started in the early 2000s and that is still ongoing. Indeed, while moving away from classical strategies that use animals to generate apical toxic outcome assumed to be extrapolatable to humans, more predictive human-based approaches devoid of animals are progressively being adopted worldwide. One of the historical milestones in this regard was the publication of the report “*Toxicity testing in the 21st century: a vision and a strategy*” by the US National Academy of Sciences in 2007, which fully embraced these novel approaches and that highlighted the importance of mechanistic toxicology (NRC, 2007). In this respect, (human-based) *in vitro* cell culture methodologies are strongly preferred over complex animal models to elucidate mechanisms of toxicological action, The term “*in vitro*” originates from Latin and literally means “in the glass.” In a toxicological context, it refers to the measurement of adverse interactions outside a normal biological context by using isolated organs, tissues, cells, or subcellular fractions (Blaauboer, 2017). Recent advances in the field now allow *in vitro* modeling within physiological contexts, such as envisaged in microphysiological systems.

It is therefore not surprising that the area of *in vitro* toxicology is gaining *momentum*. Furthermore, as holds for science in general, *in vitro* toxicology has evolved to become a multidisciplinary area. *In vitro* toxicology nowadays indeed feeds from a broad variety of fields, ranging from natural, formal and applied sciences even all up to social sciences and humanities. Frontiers in *In Vitro* Toxicology will embody these recent advancements by serving as a hub for dissemination and highly visible worldwide exchange of information regarding cutting-edge developments in *in vitro* toxicology. Frontiers in *In Vitro* Toxicology will publish peer-reviewed original research and review papers on any topic pertinent to the dynamic field of *in vitro* toxicology. The target audience includes undergraduates to full professionals in academic, industrial, and regulatory settings in any part of the world. Frontiers in *In Vitro* Toxicology will encourage submission of manuscripts related to basic and applied *in vitro* toxicology relevant to a wide spectrum of applicability domains and involving a broad chemical space, such as pharmaceuticals, food additives, cosmetics, biocides, and industrial chemicals.

## Author Contributions

The author confirms being the sole contributor of this work and has approved it for publication.

### Conflict of Interest

The author declares that the research was conducted in the absence of any commercial or financial relationships that could be construed as a potential conflict of interest.

## Footnotes

^1^Directive 2010/63/EU of the European Parliament and of the Council of 22 September 2010 on the protection of animals used for scientific purposes. OJ L276, p. 33–79.

^2^Regulation 1223/2009 of the European Parliament and of the Council of 30 November 2009 on cosmetic products. OJ L342, p. 59–209.

^3^ https://www.cosmeticsdesign-europe.com/Article/2019/03/05/Global-ban-on-animal-testing-where-are-we-in-2019

## References

[B1] Blaauboer B. J. (2017). *In vitro, ex vivo, in vivo* toxicology, the terminology issue. Toxicol. In Vitro 45, iii–iv. 10.1016/j.tiv.2017.09.028 29037367

[B2] Laverty H. G. Antoine D. J. Benson C. Chaponda M. Williams D. Kevin Park B. (2010). The potential of cytokines as safety biomarkers for drug-induced liver injury. Eur. J. Clin. Pharmacol. 66, 961–976. 10.1007/s00228-010-0862-x 20694460

[B3] NRC (2007). Toxicity Testing in the 21st Century: A Vision and a Strategy. Washington, DC: The National Academies Press, 196.

[B4] Russell W. M. S. Burch R. L. (1959). The Principles of Humane Experimental Technique. London: Methuen, 238.

